# Enhancing the Usability of an Optical Reader System to Support Point-of-Care Rapid Diagnostic Testing: An Iterative Design Approach

**DOI:** 10.2196/humanfactors.8621

**Published:** 2017-11-21

**Authors:** Jess Hohenstein, Dakota O'Dell, Elizabeth L Murnane, Zhengda Lu, David Erickson, Geri Gay

**Affiliations:** ^1^ Department of Information Science Cornell University Ithaca, NY United States; ^2^ School of Applied and Engineering Physics Cornell University Ithaca, NY United States; ^3^ Sibley School of Mechanical and Aerospace Engineering Cornell University Ithaca, NY United States

**Keywords:** telemedicine, point-of-care systems, self care, biomedical technology

## Abstract

**Background:**

In today’s health care environment, increasing costs and inadequate medical resources have created a worldwide need for more affordable diagnostic tools that are also portable, fast, and easy to use. To address this issue, numerous research and commercial efforts have focused on developing rapid diagnostic technologies; however, the efficacy of existing systems has been hindered by usability problems or high production costs, making them infeasible for deployment in at-home, point-of-care (POC), or resource-limited settings.

**Objective:**

The aim of this study was to create a low-cost optical reader system that integrates with any smart device and accepts any type of rapid diagnostic test strip to provide fast and accurate data collection, sample analysis, and diagnostic result reporting.

**Methods:**

An iterative design methodology was employed by a multidisciplinary research team to engineer three versions of a portable diagnostic testing device that were evaluated for usability and overall user receptivity.

**Results:**

Repeated design critiques and usability studies identified a number of system requirements and considerations (eg, software compatibility, biomatter contamination, and physical footprint) that we worked to incrementally incorporate into successive system variants. Our final design phase culminated in the development of Tidbit, a reader that is compatible with any Wi-Fi-enabled device and test strip format. The Tidbit includes various features that support intuitive operation, including a straightforward test strip insertion point, external indicator lights, concealed electronic components, and an asymmetric shape, which inherently signals correct device orientation. Usability testing of the Tidbit indicates high usability for potential user communities.

**Conclusions:**

This study presents the design process, specification, and user reception of the Tidbit, an inexpensive, easy-to-use, portable optical reader for fast, accurate quantification of rapid diagnostic test results. Usability testing suggests that the reader is usable among and can benefit a wide group of potential users, including in POC contexts. Generally, the methodology of this study demonstrates the importance of testing these types of systems with potential users and exemplifies how iterative design processes can be employed by multidisciplinary research teams to produce compelling technological solutions.

## Introduction

Rising medical costs and physician shortages are increasingly straining health care systems worldwide. Additionally, many resource-limited areas lack access to the laboratory equipment, facilities, and other infrastructure necessary for diagnosing, monitoring, and treating various conditions and diseases. Rapid diagnostic tests (RDTs) delivered at the point-of-care (POC) promise to help address this growing global need for portable, inexpensive assessment techniques that can be performed in nonclinical settings.

The lateral flow assay (LFA) is a widely used RDT that uses a paper-based strip to collect biological samples (eg, blood, saliva, urine, or other fluids) and measure biomarkers of interest (eg, antibodies, pathogens, and proteins). To produce a quantitative test result, the strip must be analyzed using some type of reader, which a number of commercial and research endeavors have focused on creating. However, existing solutions tend to be bulky, expensive to manufacture, or require specialized knowledge to use, making them infeasible for deployment in many POC settings. This presents a need for a reader that is portable, affordable, and highly usable.

In addition, today’s ubiquity of smart devices (eg, mobile phones and tablet computers) along with their processing power and data capture capabilities presents a broad opportunity to enhance POC medical diagnostics. The field of mobile health (mHealth) focuses on realizing this potential—for example, by developing novel systems that enable remotely located doctors or even patients themselves to use smart devices to perform clinical tests and view the results in real time. When such a test requires a sample of blood or other body fluids, a typical mHealth approach would utilize additional sensor hardware (eg, an aforementioned reader) that could analyze the sample as well as pair with a smart device to deliver the results through its interface.

The research presented in this paper focuses on the development of such hardware, presenting a novel system for biomarker-based health assessment developed by a multidisciplinary team of nutritional scientists, mechanical engineers, and human-computer interaction researchers through an iterative, usability-focused design process.

We call the final version of our device Tidbit, a stand-alone reader that can be wirelessly controlled by a smartphone or any other Wi-Fi-enabled computing system. This ability to pair with smart devices facilitates the regular installation of software updates and, in turn, more robust performance. Tidbit’s integration with smart devices also boosts processing power and employs familiar interfaces that afford intuitive interactions for end users even without significant training. The system can be deployed in various contexts, including traditional clinical settings, POC diagnostics in resource-limited locations, or as part of at-home health self-monitoring.

### Lateral Flow Assay Technology

As mentioned above, an LFA is essentially an instrument used for detecting the presence, absence, or specific level of some biological substance of interest, often referred to as an analyte or biomarker. Most LFAs do not require any external reagent, and a biological sample is simply applied to a testing medium (eg, a porous membrane that can transport fluid) to initiate and complete the test. An example of a well-recognized LFA is the home pregnancy test, which detects the presence of a hormone produced during pregnancy and displays a binary result. More broadly, the LFA has a wide range of applications, including micronutrient monitoring [1], as well as detection of diseases, internal organ failure [2], toxins [3], or illegal drugs [4]. The LFA’s compact and portable form, low production cost, near-immediate output of results, and overall ease of use make it a popular rapid diagnostic test (RDT).

LFA technology continues to advance, for example, to provide quantitative output (ie, to measure the level of a biomarker of interest in a sample rather than a binary test result of whether or not the analyte is present). This quantification of the LFA signal is necessary for early-stage, high-sensitivity, and precise diagnostics. Traditionally, quantification has been performed in a clinic or research laboratory using benchtop research-grade instruments such as microwell plate readers [5-7]. Such instruments offer very high performance, but their large size and expensive cost make them infeasible for use in more modestly resourced application areas. Development of robust, portable, and low-cost LFA reader systems is therefore imperative to support diagnostics in POC, personal, and resource-limited settings.

The specific contributions of this study are presented in Textbox 1.

**Specific contributions of this study.**

Specific contributions

- A series of functionality requirements and design considerations were identified as important to consider when developing mobile health (mHealth) technologies that support rapid diagnostic testing at the point of care
- A novel, fully functional device known as the Tidbit created via an iterative design process aimed at satisfying these specifications
- Findings and insights from multiple trials with users, including a better understanding of potential contexts of use, as well as compelling directions for future research
- A demonstration of how to go about employing iterative design methodology when creating novel mHealth hardware solutions, along with a discussion of the value of engaging in such processes

**Table 1:** Most common customer-requested features for a lateral flow assay reader and corresponding features of the new Tidbit.

User-requested features	Corresponding design goals
Ease and convenience of operation	User can use their own smart device; automatic analysis at optimal time; results displayed in easy-to-read visual; can be powered by internal rechargeable battery or alternating current adapter
Quantitative read out	High accuracy imaging algorithm; result output in numbers with units or qualitatively
Automatic electronic documentation of results	Saves data on user's smart device; results can be sent to physician
Higher sensitivity	Highly optimized sensor; ability to incorporate fluorescence and other detection labels
Objective interpretation of results	Raw image data stored
Use of reader in quality control for strip manufacturing	Rapid test time allows high throughput; stand-alone portable reader; automatic checks for test strip validity
Handheld format and mobility	Small format reader; portable
Operational robustness	Repeatable testing process; protected data; automatic checks for result validity; data assigned a time, date, and patient ID
Physical robustness	Can be used in any lighting conditions
Audio or visual display of results	Visual display of progress and results
Connectivity to data management system	Wireless connectivity to any Wi-Fi-enabled device
Hard copy of test results	Stored results can be shared and printed
Compatibility with clinical workflow and systems	Easy incorporation into clinical systems
Stand-alone reader	Rechargeable battery power; works with any smart device
Batch/calibration data management system	Automatically builds secure database of results on smart device being used
Low price	Integrated flexibility—any cassette format can be used and any detection label can be read
Appealing design	Convenient shape and feel; trendy and professional design
Fast read out	Imaging and analysis takes only a few seconds
Wireless data transfer	Standard feature
Compatibility with unique cassette format	Standard feature
Compatibility with unique label	Ability to incorporate fluorescence and other detection labels
Available professional software	Standard feature
Compatibility with different tests and formats	Standard feature
Multiplexing	Can analyze multiplex signals

### Designing Lateral Flow Assay Reader Devices

Several research and commercial endeavors have investigated smartphone-based reader systems that take advantage of the universal familiarity and ubiquity of smartphone technology. These systems typically include an accessory that attaches to a smartphone and often rely on the phone for imaging and quantifying results. Some technologies, such as the HRDR-300, feature an optomechanical smartphone attachment that reads fluorometric LFA [8], whereas others feature an optomechanical smartphone attachment that reads colorimetric LFA [1,9]. The Gene-Z [10] performs genetic analysis through a large iPod attachment and a custom microfluidic chip. Others use electrochemical sensing smartphone attachments integrated with custom microfluidic chips [11], whereas another employs an optomechanical attachment to turn the smartphone camera into a microscope for sample imaging [12]. Despite the promise of these technologies, each has limited functionality and potential applications. The systems discussed have been developed to perform with specific test formats and are only compatible with specific smartphones, resulting in limited potential applications and markets. Additionally, even with an eventual goal of being a consumer product, none of the discussed technologies have addressed the principles of universal design or usability.

Beyond accurate performance, a number of features that users consider important and should be included in an LFA reader have been identified in the literature, as shown in Table 1 [13]. Unfortunately, our review of the aforementioned systems finds that current readers do not fulfill many of these requirements, particularly those regarding simple operation, mobility, speed, and low cost.

## Methods

This study addresses the above motivations and goals through an iterative design methodology. Specifically, the research consists of three stages and accompanying system variants: (1) a design critique and in-lab study with 26 participants of our germinal prototype NutriPhone; (2) ideation, prototyping, and in-lab evaluations with 6 participants of the second version of our system that adopted a stand-alone reader; and (3) development and in-lab evaluations with 6 participants of our refined reader system Tidbit.

### Version 1: The NutriPhone System

#### System Specification

**Figure 1:**
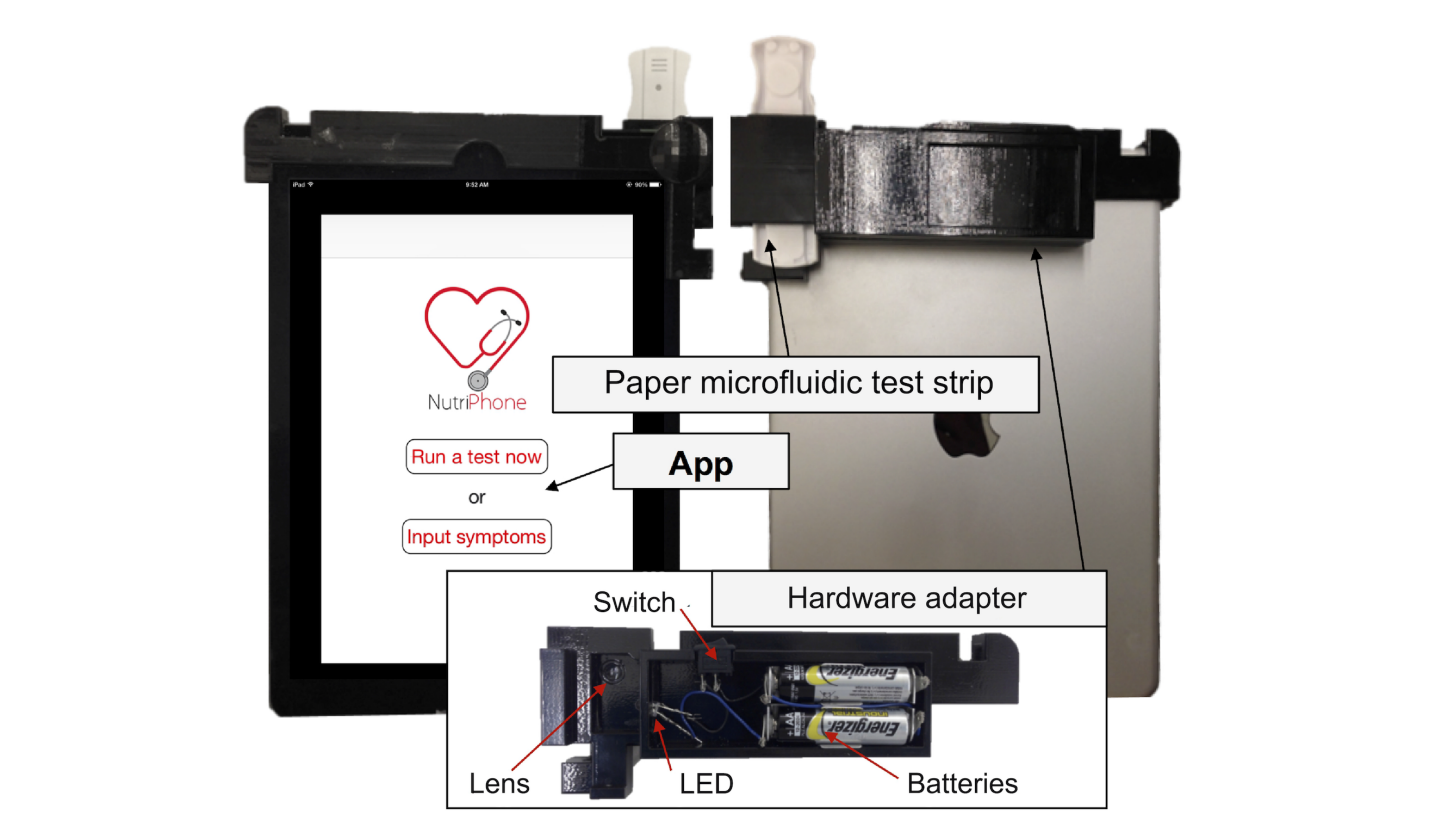
The Version 1 system consists of a plastic accessory that clips around an Apple iPhone or iPad, a disposable lateral flow assay test strip, and an app that processes images and displays results.

Our initial ideas were rooted in the design space of prior mHealth diagnostic systems, leading us to first focus on developing reader hardware that could attach directly to a smartphone. We called our first version NutriPhone, which, as shown in Figure 1, consists of a small plastic reader accessory that clips over a smartphone’s camera, a custom LFA in a plastic cassette, and a smartphone app that guides the user through the testing process and delivers the diagnostic result.

To use the system, a user starts the app on an Apple iPhone or iPad and is presented with step-by-step instructions for testing the analyte of interest. This process involves a finger prick to collect a single blood droplet on the test strip, which is then inserted into the clip-on attachment. The software takes a picture of the test strip using the phone or tablet computer’s camera, performs the appropriate analysis, and displays the result to the user. The entire process, including the blood draw, takes approximately 10 to 15 min.

#### Team Design Critique

Our first step in evaluating the design of the NutriPhone prototype was an in-house critique informed by the LFA and usability literature. We identified several areas for improvement related to software compatibility, physical specifications, and contamination.

First, similar to a number of existing reader systems, our Version 1 (V1) NutriPhone is only compatible with iOS devices. However, Android is currently dominating the smartphone market with a worldwide smartphone market share of 87.6% [14], resulting in a very limited market for NutriPhone among global smartphone users. Furthermore, considering that a key POC application area is resource-limited settings where many people do not own a smartphone [15], we see a need to move toward a platform-agnostic reader.

In addition—and again similar to many existing readers—our V1 prototype will not fit over a case or cover that may be on the user’s smartphone or tablet computer. A different design is therefore necessary to accommodate the various physical constraints of today’s smart devices. Relatedly, the physical specifications of readers such as NutriPhone typically fit only one particular test strip cassette size and shape, requiring a multitude of apparatus to read test strips of different types from diverse manufacturers. Finally, we recognized that contamination could be a major problem with current readers, including NutriPhone, as the user is required to place body fluids directly adjacent to a smart device.

#### In-Lab User Studies

We next held institutional review board (IRB)-approved (Protocol ID# 1410005065) in-lab human trials with 26 participants to assess basic usability and receptivity toward NutriPhone. Participants included 20 females who were drawn from an on-campus recruiting system and were aged between 18 and 27 years. Participants had varying levels of education, ranging from high school to graduate degrees. None had previously used our device.

Each participant was given information about the function of the test strips and the general purpose of the reader. They were not given any technical background on how the reader works or how to use it, as we wanted to observe each participant figuring out the process on their own. For this study, we paired the reader with an Apple iPad 2 and had the app open and ready to use. Without instructions, each participant ran through the steps of the app and eventually placed a prerun test strip into the reader, which generated a predetermined result. The participants were informed beforehand that this result was randomly generated and did not reflect their own personal medical data. We collected observational notes, encouraged participants to think aloud, and conducted interviews where we asked questions about the device and use process. The entire process took less than 30 min. Although the size of our study sample was limited, we were able to identify a number of features and use cases that we would need to support in future design iterations.

To begin, all participants had some level of difficulty finding the power switch and test strip insertion point, causing them to flip or rotate the Apple iPad and attached device in an attempt to locate these components, suggesting that the design was unintuitive. Additionally, 6 participants expressed hesitation about inserting the test strip into the device, and we observed their uncertainty about which orientation to use to insert the strip as well as their failure to insert the strip all the way. Similarly, several participants honed in during interviews on the “confusing” nature of inserting the strip.

Our qualitative thematic analysis of the interview data surfaced more encouraging themes as well, particularly regarding the “very easy” usability of the app. One of the participants stated that s/he:

...liked how the text was really big and the instructions were fairly clear; it came with images.

Participant 9

Another participant said:

...there are visual instructions, so for [inexperienced] people or for the elderly, it’s easy to maneuver.

Participant 10

Altogether, our internal critique and user study motivated us to preserve the positively received components of NutriPhone’s app interface and focus our next design iteration on addressing the identified limitations and problems of the V1 hardware, such as compatibility, contamination, and overall usability.

### Version 2: A Stand-Alone Reader

In accordance with the increasing focus on usability in product development and case studies reporting on its practical implementation [16], we undertook iterative design methods to create an updated system aimed at addressing the shortcomings of the initial version as well as the current state-of-the-art LFA reader technology.

#### Design Ideation

On the basis of identified problems with the current readers, user-requested features, and participant feedback from our V1 prototype, we settled on a number of crucial characteristics for the next version of our reader. These specifications allowed us to immediately make several decisions regarding the format and components of the new design.

Paramount was simple operation, which was not observed with the V1 prototype, along with mobility and low cost, which are particularly important for POC diagnostics in resource-limited settings. To allow total mobility, we wanted to create a reader that was battery-powered, and to support universal compatibility, we opted for a stand-alone reader that would not require physical attachment to the smart device controlling it. To keep the cost low, we aimed for a design with minimal materials and parts, which resulted in an approximate cost of US $60 for our prototype. Assuming that mass production could bring the cost down even further, this makes our device much less expensive than currently used lab equipment such as microplate readers, which cost thousands of dollars, and other POC readers, which are typically priced in the hundreds of dollars [10].

To increase portability and usability, we also found it important to make the new reader compatible with any smart device and incorporate the ability to read test strip cassettes of any size and LFA format (eg, multiplex and different detection labels). We also considered the eventual incorporation of different filters into the reader, which will be necessary to read different detection labels. Additionally, a static external camera was employed to overcome the technical limitations and inconsistency involved in using a smartphone camera for imaging.

Lastly, we emphasized the creation of a unique and appealing design that evokes positive affect to promote adherence and individuals’ overall desire to continue long-term use of the device in an at-home health monitoring setting. To increase the reader’s ease of use, we decided to make the internal components entirely enclosed so that the user’s only concern would be to place a test strip into the reader without becoming distracted or overwhelmed. We therefore incorporated a pull-out tray for insertion of test strips. We also placed indicator light-emitting diodes (LEDs) on the outside of the reader to let the user know that the system was functioning correctly.

#### Prototyping

**Figure 2:**
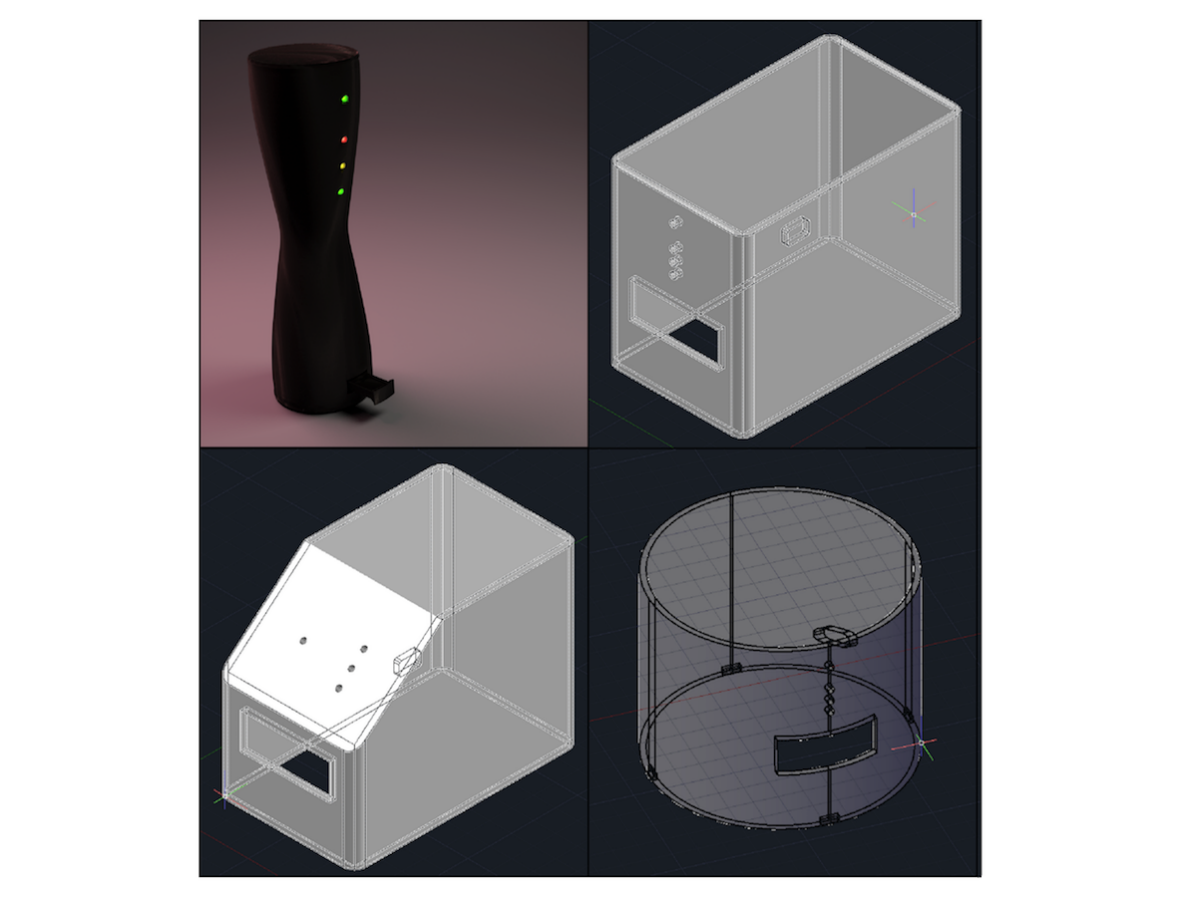
Clockwise from top left: rendering of hourglass design, computer-aided drawing of rectangular prism, cylinder, and rectangular prism with sloped face designs. All incorporate a pull-out tray for insertion of test strips, external indicator light-emitting diodes, and a charging port.

On the basis of these criteria and the requisite dimensions, we imagined various design forms, the first of which was an hourglass shape as shown at the top left of Figure 2. However, although the design received positive reception from our research team and other colleagues during informal feedback sessions, we soon abandoned the hourglass reader upon realizing its impracticality with regard to housing the necessary internal components. Other ideas, also shown in Figure 2, included a simple rectangular prism, a rectangular prism with a slanted front face, and a cylinder. Both rectangular prism designs offered simplicity, with the slanted face variant’s asymmetry reducing the risk of users attempting to place the reader upside down. Inspired by recent devices such as the Amazon Echo [17], Mac Pro [18], and Google OnHub [19], the cylinder design added visual interest with a compact physical footprint.

**Figure 3:**
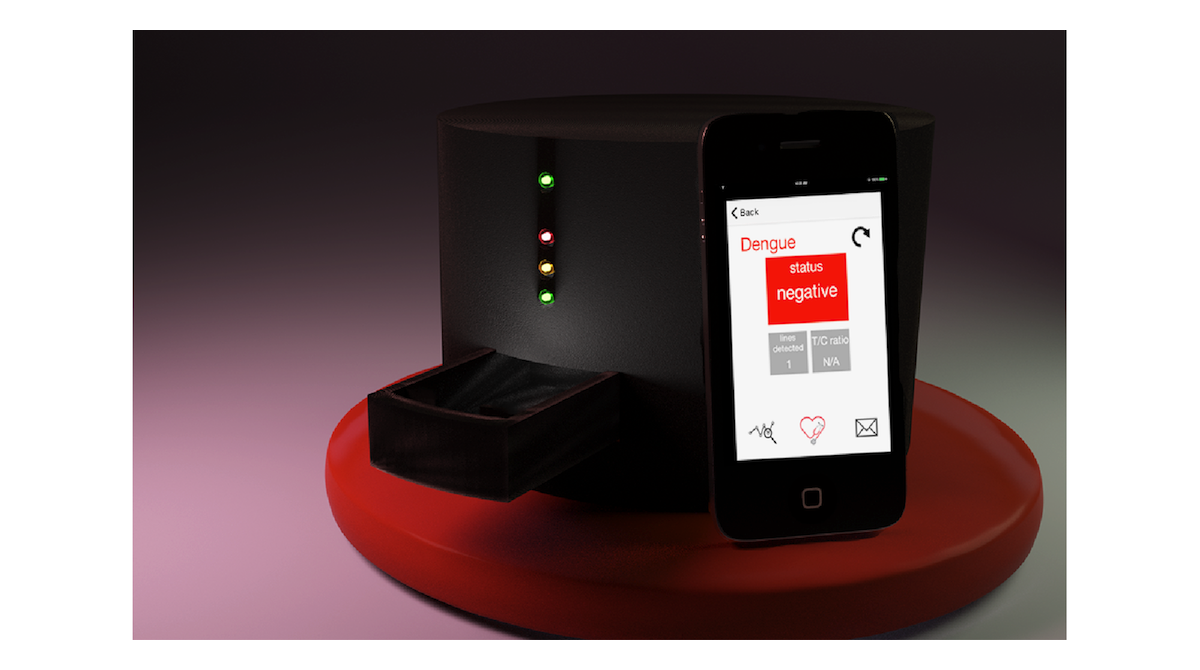
Rendering of the Version 2 design with Apple iPhone for size reference.

Three-dimensional printed prototypes of the designs were printed in white acrylonitrile butadiene styrene plastic and painted black. Weighing a number of trade-offs related to fabrication complexity, aesthetic appeal, and physical footprint, we ultimately settled on moving forward with the cylindrical design as rendered in Figure 3.

**Figure 4:**
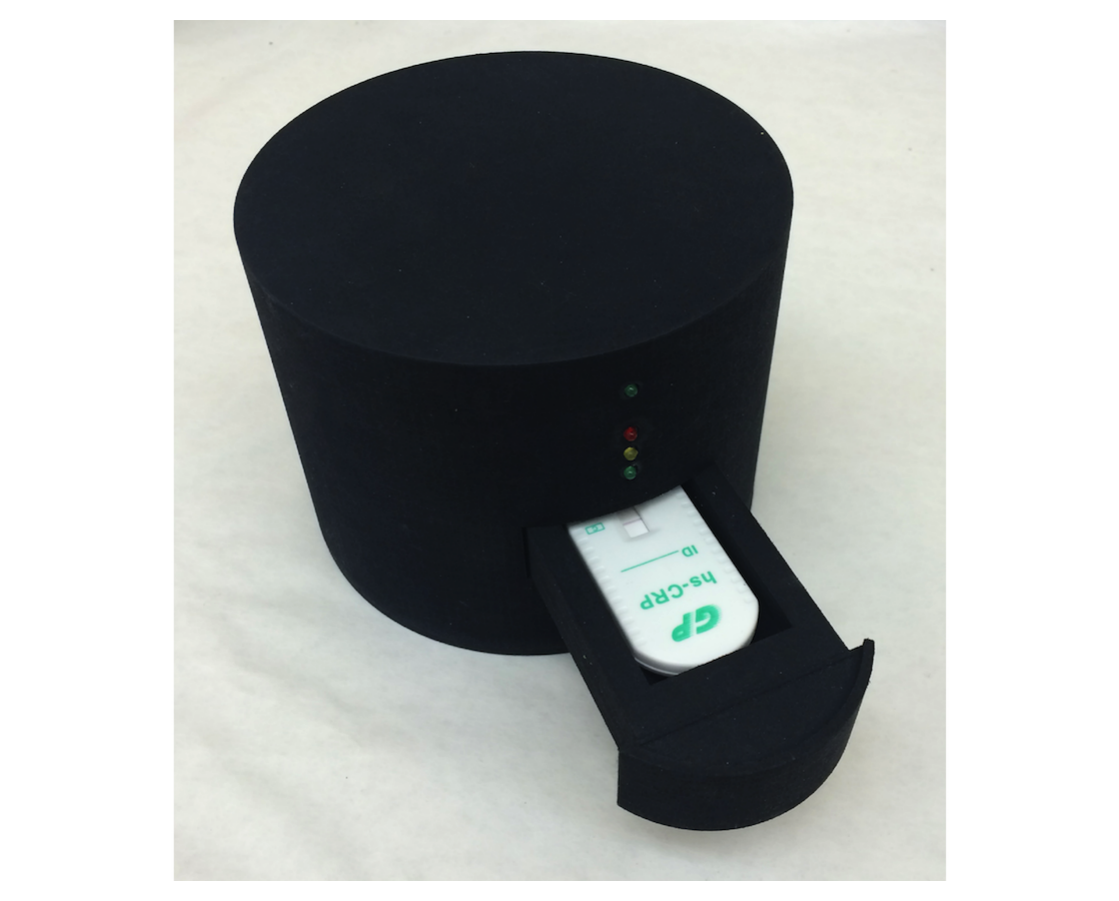
Version 2 prototype with a large format C-reactive protein test strip cassette inserted in the pull-out tray.

Figure 4 shows the high fidelity, assembled Version 2 (V2) prototype. To support testing consistency and repeatability, the case is made of opaque plastic, which isolates the internal components and LFA from variable external light. This also allows the device to be used in any lighting conditions, from bright sunlight to total darkness, without any loss of image quality. The pull-out tray also supports consistency and repeatable imaging, as the tray’s design accommodates various test cassettes from diverse manufacturers, and the edges of the tray are sloped so that variably sized cassettes are held securely in the center.

For diagnostic testing, a user first powers on the reader, running it on either alternating current (AC) power or the battery. The reader then broadcasts a preconfigured encrypted wireless network, allowing direct wireless transfer of commands and image data to a smartphone, tablet computer, computer, or other Wi-Fi-enabled device. This approach also allows for additional security, as the reader can only be accessed by a device that is in close proximity and has the necessary credentials.

After the reader and device are connected, the user can begin a diagnostic test by launching the system app. Because the reader works with various types of LFA, the app first asks a user to select the desired test from a list of options. This selection loads the corresponding procedure, which varies between test type in terms of time required between steps, the region of interest for image analysis, and the calibration curve used to quantify analyte concentration. The app then shows a series of steps and pictures that instruct the user in performing the appropriate sample collection and strip cassette insertion for the chosen test. The usability of these instructions was verified in the V1 in-lab study.

**Figure 5:**
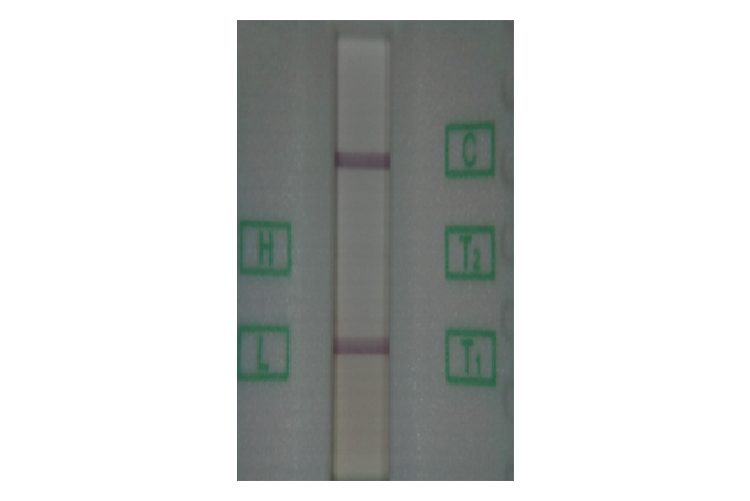
An image of test and control lines on a lateral flow assay taken by our Version 2 reader shows consistent, flat illumination.

Once a test cassette is inserted into the reader, indicator LEDs on the outside of the reader show development and analysis progress in the form of a stoplight-style red-yellow-green lighting system. When the test strip has completely developed, the internal lighting and camera are activated to capture an image of the strip (Figure 5), which is then sent to the smart device. The software then performs the necessary image analysis and compares the result to a predetermined calibration curve to obtain a quantitative result, which is displayed to the user and stored in the app. No results are stored on the reader itself, meaning it does not contain any sensitive patient data and can be used asynchronously by multiple users without risking the disclosure of individual results.

#### In-Lab Usability Testing

To assess usability and potential use cases and to gather general feedback and insights, we ran an IRB-approved (Protocol ID# 1602006140) small-scale usability study with 6 participants (3 females, 3 males, aged 22-72 years) recruited by word of mouth. All participants were native English speakers residing in the northeastern United States, and they had varying levels of education, ranging from some high school to graduate degrees. None had previously used our device.

Each participant was first given information about the function of the test strips and the general purpose of the reader. They were not given any technical background on how the reader works or how to use it, as we wanted to observe each participant figuring out the process on their own. For this study, we paired the reader with a laptop computer and had the app open and ready to use. Without instructions, each participant ran through the steps of the app and eventually placed a prerun test strip into the reader, which generated a predetermined result. That is, no blood was drawn, given that our goal was focused more on exploring how participants feel about and handle interacting with the system. The participants were informed beforehand that this result was randomly generated and did not reflect their own personal medical data. After receiving the result, each participant was asked to complete a System Usability Scale test, and the study then concluded with an interview session to gather open-ended feedback about the V2 system and the testing process. The entire process took less than 30 min.

The resulting mean system usability (SU) score was 87.1 (σ=9.8), which is above the 90th percentile when compared with prior scores of other systems [20]. Although the sample size was small (n=6) and additional research would be necessary to confirm whether these results generalize to wider populations, they do provide a solid preliminary indication that different age groups and education levels found our V2 design to be highly usable.

The feedback given in the debrief interviews and our observations during participants’ interactions with the system were also encouraging. One of the most noteworthy findings was that the older participants (n=3, aged 65-72 years) generally expressed much more excitement about the new reader and the process of using it than the younger participants (n=3, aged 22-26 years), reinforcing the need for a universal design. Multiple participants (n=3) also highly appreciated the battery power and wireless aspects of the reader, and most (n=4) either explicitly commented on the helpfulness of or could be observed reacting positively to the external indicator LEDs. In addition, all participants were able to intuitively handle simultaneous use of the app in conjunction with the reader, affirming our assumption that incorporating a familiar device would aid the usability of a new system.

However, most participants (n=5) expressed confusion, either verbally or through facial expressions, about which way the test strip should be inserted into the reader. In fact, the process of placing the test strip into the reader universally caused the most hesitation among all participants. One participant thoroughly examined the reader and wondered where the test strip should be inserted before realizing that there was a pull-out tray. Without prompting from us, the participant went on to explain the confusion by saying:

[It]’s hard to see with all the black. Maybe you should write “PULL” on it.

### Version 3: The Tidbit

#### Design and Development

On the basis of insights gained in the evaluation of V2, we further refined the design of our reader, which we called Tidbit. The internal components, cost, and functionality of Tidbit are the same as the V2 design, as our focus in this iteration was on enhancing user interaction.

**Figure 6:**
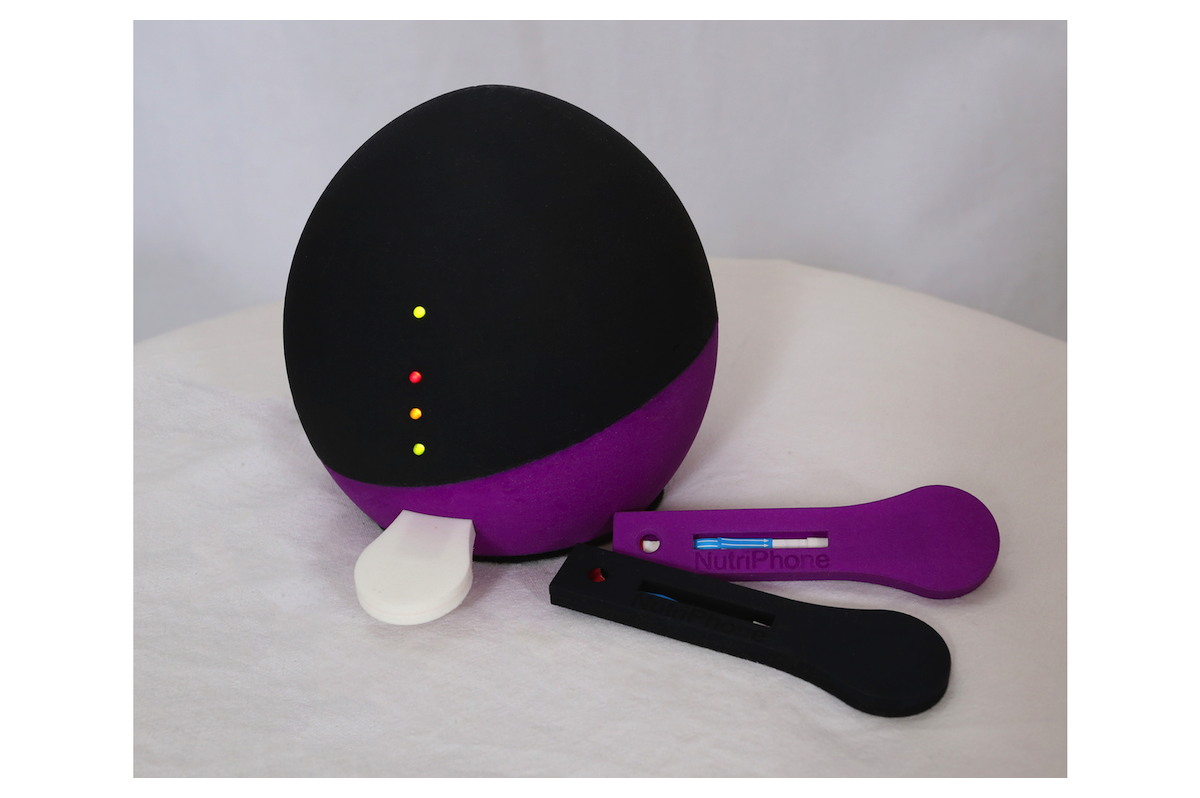
Tidbit, our Version 3 design and new test strip cassettes. The confusing pull-out tray has been eliminated, and the asymmetry of the device prevents users from inadvertently placing it upside down.

As seen in Figure 6, the new reader features a novel egg shape, which elicited positive reactions from potential users during informal feedback sessions. This design is also no longer symmetrical, reducing the chance of a user inadvertently placing the Tidbit upside down. Tidbit is also smaller than the V2 design, making it more portable and less expensive to produce. Additionally, the pull-out tray has been eliminated, and the test strip cassette is now inserted directly into the reader. This feature was aimed at eliminating the confusion we observed regarding where the strip should be inserted. To combat any additional confusion, we also added more detailed pictures in the app instructions that explicitly illustrate which way the test strip should be inserted. Finally, Tidbit features two colors that could be changed to suit the end user’s needs or tastes.

#### In-Lab Usability Testing

Undertaking a round of evaluations on our Version 3 (V3) system, we again conducted a small-scale IRB-approved (Protocol ID# 1602006140) study to assess usability and gain feedback from potential users. By word of mouth, we recruited 6 participants (3 male, 3 female, aged 24-54 years) who all reside in the northeastern United States and have varying levels of education, ranging from high school to graduate degrees. None had previously used our device. The study’s procedure was identical to that of the V2 usability trial, except the Tidbit was wirelessly paired with a tablet computer (Apple iPad 2), rather than a laptop computer.

## Results

Overall, the study indicated that the Tidbit’s design changes made the device more intuitive and straightforward to use. We saw a bump in mean SU score to 89.6 (σ=7.3), which, as mentioned with the V2 system, is above the 90th percentile when compared with scores of other systems [20]. In addition, all participants were able to intuitively handle simultaneous use of the Apple iPad app in conjunction with the V3 Tidbit reader, which, together with our similar findings from the V2 study with a laptop computer, suggests Tidbit’s ease of use across a range of platforms.

Though minimal, participants did express a few suggestions for even further improvement that are worth noting as design considerations for future study in this and related areas. The first issue surfaced because Tidbit was placed on a table or desk below participants’ direct eye level, and so the device’s curved face seemed to make the test strip insertion point difficult to see for some. A few participants (n=2) experienced momentary trouble in finding the slot and used their hands to physically search for and moved their heads downwards to look at the device head on and find the slot. One participant summarized this concern:

The hole was hard for me to see immediately. Maybe it could be moved up.

The other main insight we noted as highly worthwhile to pursue going forward relates to delivering visual or other forms of feedback “that [the device] is working throughout,” as one participant put it. For a device such as Tidbit, this may be accomplished by adjusting the timing of the indicator LEDs on the reader or by integrating additional feedback about the connection status and test strip development progress into the app interface.

## Discussion

### Principal Findings

“In analytical fields that pride themselves on scientific basis and experimental rigor, the hidden danger is to neglect areas that are not easily addressed in the framework of science and engineering” [21]. It is now well recognized that engineers can easily forget that they are not the typical users of the technologies they build, leading them to make development decisions that are not well suited to their target populations or contexts. This is an understandable challenge, given that experts’ extensive specialized knowledge of a system makes it difficult to have empathy for and fully understand the needs of an end user; however, it is quite problematic in practice, as such a user-detached approach is likely to hamper adoption, adherence, and ultimately, impact. In this study, we have demonstrated the process and illustrated the value of utilizing iterative techniques and focusing on usability, providing an example of how multidisciplinary teams can successfully implement these design methodologies. The iterative design process and focus on usability detailed in this study informed a significant shift in design specifications for our system and revealed design considerations that could extend to the development of other mHealth technologies.

### Design Consideration 1: Design Features for mHealth Technologies Should Adhere to the Principles of Universal Design and Accessibility

In developing systems with a breadth of possible use settings including clinical, POC, and resource-limited, it is essential to balance the needs of diverse users and create a design that is easily accessible for potential user communities. Through qualitative analysis of participants’ comments, researchers’ observations, and participants’ interviews, we were able to improve the accessibility of our designs and hone in on elements that were helpful to users.

Specifically, in terms of software user interfaces for mHealth apps, we found that a large, black font on a white background was viewed as a vital element for accessibility among a diverse population. Similarly, large navigation buttons and straightforward, simple language seemed to aid users in progressing through the app instructions. We also observed the enormous benefits of visual aids, including diagrams, photos, and video, in addition to the textual instructions, as many users commented on their helpfulness in guiding them through the procedure. Although participants in our usability trials were native English speakers, such visual aids could also be beneficial for users who are less familiar with written English.

With regard to hardware for POC health technologies, we recommend designing platform-agnostic systems whenever possible. In addition to allowing universal compatibility in diverse settings, not relying on a particular smart device allows designers to take advantage of users’ familiarity with their own personal device. We also propose that POC technologies are developed to be low cost, wireless, and battery-powered, as users noted the importance of these features, and economical pricing and portability are particularly important for adoption in resource-limited settings where budget and reliable infrastructure (eg, electricity and transportation) can be problematic.

### Design Consideration 2: Simple Operation and an Appealing Design Could Help in System Adoption and Adherence

In designing mHealth systems for long-term adoption, encouraging continued use is essential. This goal inherently implies the importance of a simple, intuitive design and a rewarding experience—essentially, users need to enjoy using it. With our V1 prototype, we observed that this was not the case, as users struggled with various aspects of the hardware, and we addressed the observed issues, along with any new ones that arose, in our subsequent design iterations. The resulting Tidbit design is asymmetrical with external lights indicating that the reader is correctly functioning, which have been shown to enhance the overall usability of the system. Through both qualitative and quantitative analyses, we verified that our final Tidbit design is both well liked and easy to use. Although long-term adherence needs to be verified in future field trials, we hope that our attention to simplicity and aesthetics in our design will aid in continued use.

### Design Consideration 3: Remain Flexible During the Design Process

The incorporation of universal design principles through an iterative design process implies that designers will likely need to rethink their ideas and make alterations whenever necessary. After considering user needs (Table 1), our findings from the user trials with the V1 prototype, and the goal of adhering to universal design principles, we realized that a complete overhaul of our original ideas was necessary. Being flexible in our design process allowed us to abandon the conventional smartphone attachment design that has been used in the previously discussed reader technology [1,8-12] and imagine a novel stand-alone reader. A stand-alone reader solves many issues inherent to smartphone-attaching readers, including variability in image quality and processing power with different smartphones, compatibility with various smartphone types and sizes, and placing biomatter near a user’s personal smartphone.

### Limitations and Future Work

In addition to potential future work we mentioned previously (eg, rethinking positioning of the test strip slot and delivery of more visual feedback), a key next step is conducting field trials of Tidbit in naturalistic at-home, remote clinical, or other POC contexts to ensure that it remains an effective and usable tool in realistic settings and over extended periods of usage. One particular concern is that participants in lab-based studies such as ours can display demand characteristics [22] wherein they try exceptionally hard to perform well at the given task, so results could be different in the wild.

Another limitation of our in-lab evaluations was their small sample sizes, although 5 participants are typically considered sufficient for usability studies [23]. Additionally, restrictions on our time and budget did not allow us to test our designs with more diverse samples, such as resource-limited populations. It would therefore be desirable to involve larger and more diverse groups of participants in studies such as ours, going forward.

Additionally, the goal of our usability studies was to examine how participants used the new hardware design, so participants did not go through the process of pairing the Tidbit with the computing device. This would essentially involve powering on the Tidbit and connecting a smart device (eg, laptop computer, smartphone, etc) to the Tidbit wireless network, so, in theory, anyone who has connected a device to a wireless network should be able to complete this step. However, future usability studies should investigate how easy it is for participants to do this, as it will be very important if the system is going to be marketed as an in-home product.

### Conclusions

This study presents the design process undertaken to develop the Tidbit, an inexpensive, easy-to-use, portable optical reader for LFA signal quantification. Potential applications for our system include in-home personal health monitoring; use in a clinic, doctor’s office, or pharmacy; and diagnostics in resource-limited settings. Our iterative design methodology enabled us to derive a novel, robust technological solution grounded in the preferences and identified requirements of potential users, with usability testing confirming the reader’s ease of use and overall positive reception. As researchers continue to develop portable personalized medical systems, it is important to incorporate the needs of and test with potential users throughout the design process. We hope that our study will encourage other researchers to incorporate similar approaches to create innovative systems that support the principles of universal design.

## Abbreviations

AC: alternating current
LFA: lateral flow assay
LED: light-emitting diode
mHealth: mobile health
POC: point-of-care
RDT: rapid diagnostic test
SU: system usability
V1: Version 1
V2: Version 2
V3: Version 3
